# Reproductive resilience but not root architecture underpins yield improvement under drought in maize

**DOI:** 10.1093/jxb/erab231

**Published:** 2021-05-25

**Authors:** Carlos Messina, Dan McDonald, Hanna Poffenbarger, Randy Clark, Andrea Salinas, Yinan Fang, Carla Gho, Tom Tang, Geoff Graham, Graeme L Hammer, Mark Cooper

**Affiliations:** 1 Corteva Agriscience, 7250 NW 62nd Ave, Johnston, IA 50310, USA; 2 Phenotype Screening Corporation, 4028 Papermill Road, Knoxville, TN 37909, USA; 3 University of Kentucky, 1100 Nicholasville Rd, Lexington, KY 40546, USA; 4 The University of Queensland, Queensland Alliance for Agriculture and Food Innovation, Centre for Crop Science, Brisbane, QLD 4072, Australia; 5 CSIRO Agriculture and Food, Australia

**Keywords:** Drought tolerance, genetic gain, maize, reproductive resilience, root systems architecture, water use

## Abstract

Because plants capture water and nutrients through roots, it was proposed that changes in root systems architecture (RSA) might underpin the 3-fold increase in maize (*Zea mays* L.) grain yield over the last century. Here we show that both RSA and yield have changed with decades of maize breeding, but not the crop water uptake. Results from X-ray phenotyping in controlled environments showed that single cross (SX) hybrids have smaller root systems than double cross (DX) hybrids for root diameters between 2465 µm and 181µm (*P*<0.05). Soil water extraction measured under field conditions ranged between 2.6 mm d^–1^ and 2.9 mm d^–1^ but were not significantly different between SX and DX hybrids. Yield and yield components were higher for SX than DX hybrids across densities and irrigation (*P*<0.001). Taken together, the results suggest that changes in RSA were not the cause of increased water uptake but an adaptation to high-density stands used in modern agriculture. This adaptation may have contributed to shift in resource allocation to the ear and indirectly improved reproductive resilience. Advances in root physiology and phenotyping can create opportunities to maintain long-term genetic gain in maize, but a shift from ideotype to crop and production system thinking will be required.

## Teaser

Emerging opportunity to continue long-term genetic gain in maize yield in the US corn belt by improving the balance between canopy, root, and reproductive growth and development.

## Introduction

Changes in root system architecture (RSA) were implicated in the determination of long-term yield improvement of maize in the US corn belt. Hammer *et al.* (2009) proposed the hypothesis that long-term changes in RSA in maize resulted in deeper root systems, and increased water capture and yield response to plant population density. This hypothesis is consistent with the observation that canopy temperature decreased with increasing year of commercialization in a set of hybrids grown under water deficit (WD; Barker *et al.*, 2005), and simulation of breeding strategies for improved drought tolerance (Messina *et al.*, 2011). Soil water extraction measurements among single cross (SX) hybrids commercialized between 1963 and 2009 showed no correlation of water extraction, year, or commercialization with yield (Reyes *et al.* 2015). Because the older double cross (DX) maize hybrids used prior to the 1960s are genetically and phenotypically more diverse than SX hybrids, it is possible that the shift in RSA, water extraction, and yield occurred prior to the 1960s.

In the USA, long-term crop improvement in temperate maize has resulted from pedigree breeding combined with reciprocal recurrent selection to improve hybrid performance (Duvick, 2001), and the optimization of agronomic practices such as planting density (Duvick, 2005; Cooper *et al.*, 2014b; Assefa *et al.*, 2018). Long-term gains have been demonstrated in irrigated, drought, and rainfed conditions (Cooper *et al.*, 2014a; Adee *et al.*, 2016). Beginning in the 1920s to 1930s, breeders used DX hybrids to exploit hybrid vigor and economically produce sufficient high-quality seed for farmers to plant at scale (Duvick, 2001). This process was replaced by SX hybrids in the 1950s to 1960s, when more productive inbred lines resulted from the breeding efforts. The finding that genetic gain is greater at higher plant population densities (Duvick, 2005) suggests that breeding has led to maize genotypes with greater tolerance to stress. The greater stress tolerance may be attributed to increased resource capture and/or enhanced reproductive resilience, but the relative importance of these two factors is unknown.

**Fig. 1.**
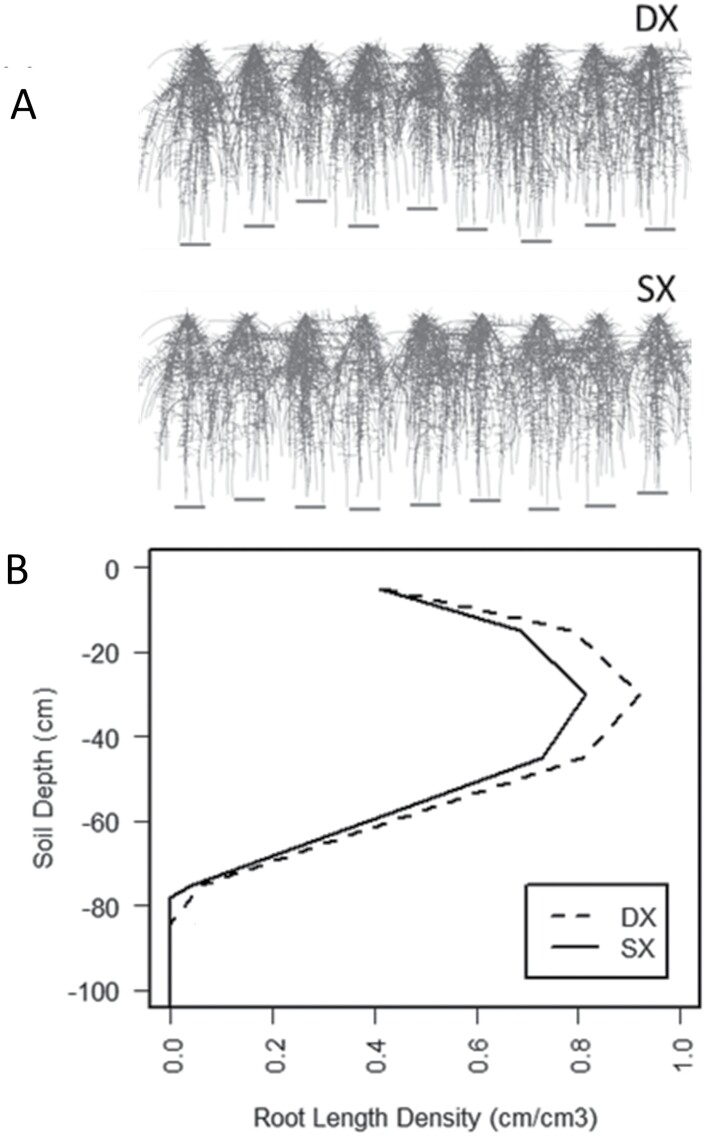
Simulated root system architecture for double (DX) and single cross (SX) maize hybrids accounting for plant to plant variation in size (A), and corresponding simulated root length densities by soil depth (B). Simulations were conducted using Corteva Agriscience proprietary software and visualized using ParaView (Kitware, NY, USA).

As elucidated in Darwinian agriculture theory, a common plant adaptation to cultivated systems is the reduction of intraspecific competitive ability (Denison *et al.*, 2003). Because of genetic segregation and fewer cycles of selection, higher intraspecific competition, emergence of stratified plant sizes, and low-yielding dominated plants (Daynard and Muldoon, 1983) are expected in DX hybrids, but to a much lower degree in SX hybrids. The more genetically and phenotypically uniform SX germplasm can produce deeper and more uniform root systems than DX germplasm, where small plants contribute root mass only in the top soil horizons. This population-emergent phenotype of RSA can influence patterns of water uptake in a manner consistent with the hypothesis proposed by Hammer *et al.* (2009). Simulations of plant populations that account for plant to plant variation in RSA show greater root length density in the top soil horizons in DX relative to SX hybrids (Fig. 1A, B). Taken together, theory, model simulations, and observations of maize performance under drought stress suggest that populations of SX hybrids capture water better than populations of DX hybrids because of lower intraspecific competition, but this has not been experimentally demonstrated.

The consequence of the root phenotypes on crop water status and drought tolerance depend on the capacity of the root system to supply the water demand established by the crop leaf area (van Oosterom *et al.*, 2016) and reproductive resilience expressed in the germplasm (Messina *et al.*, 2011). Simulations of breeding strategies suggest that expression and contribution of traits to drought tolerance are conditional on each other, and their evolution can vary with cycles of selection (Messina *et al.*, 2011). Understanding past changes in RSA and reproductive resilience can help identify opportunities to hasten genetic gain in the future. The objectives of this study were (i) to characterize RSA and soil water extraction in DX and SX maize hybrids; (ii) to test for an association between long-term changes in water extraction and RSA as postulated by Hammer *et al.* (2009); and (iii) to test the hypothesis that long-term genetic gain in temperate maize was in part determined by an increased capacity for soil water extraction.

## Materials and methods

### Characterizing RSA in controlled environments

Root phenomics for RSA were conducted on a sequence of ERA maize hybrids (Table 1) at the Phenotype Screening Corporation (PSC) in Knoxville, TN (Experiment 1). Plants were grown in hydroponic conditions using a modified Hoagland solution (241 ppm N, 10.5 ppm P, 170 ppm K, 30 ppm Ca, 55 ppm Mg, 64.5 ppm S, 0.032 ppm B, 0.12 ppm Cu, 13 ppm Fe, 0.88 ppm Mn, 0.025 ppm Mo, 0.767 ppm Zn). Maize seeds were pre-germinated and transplanted after 6 d. Phenotyping was conducted at stages V6 and V8 for RSA traits and plant height to the highest fully formed collar. Each plant container was made of fused expanded polystyrene with internal dimensions of 1000×45×200 mm. The container walls were gas permeable and allowed gas exchange throughout the depth of the container. The containers were filled with expanded polystyrene beads (Alliance Foam Technologies, Centralia, MO, USA, T180F, 42 g cm^–3^) as the growth substrate. Each container was placed in structural pods that held eight plants. The dripper assembly system for each container consisted of four equally spaced pressure-compensated dripper heads (Netafim Irrigation Inc., Fresno, CA, USA, model 01WPCJL2-B, 0.5 gallons h^–1^) operating on a 20/270 s on/off cycle at ~1.5 l h^–1^. A bank of metal halide lamps provided 400 µmol m^–2^ s^–2^ illumination on a 14/10 h day/night cycle. The temperature regime was 35 °C/24 °C for the day/night cycle.

**Table 1.** List of single and double cross hybrids and year of commercial release by experiment

Hybrid name	Cross type	Year of c ommercialization	Experiment	
			1	2
351	Double	1934	x	
322	Double	1936	x	
317	Double	1937		
340	Double	1941	x	
344	Double	1945		
352	Double	1946	x	x
347	Double	1950		
301B	Double	1952	x	x
3394	Single	1991	x	
33G26	Single	1998		
33P67	Single	1999	x	
34G13	Single	2000		
33R77	Single	2001		
33D11	Single	2005	x	x
35A52	Single	2010	x	
P1151HR	Single	2011	x	x

**Fig. 2.**
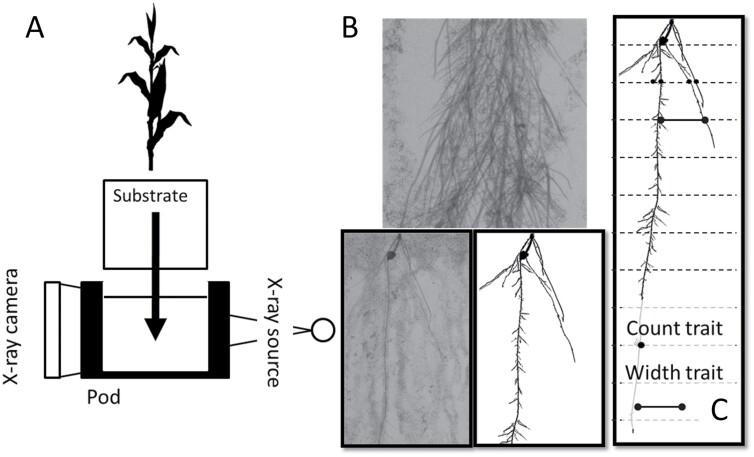
Low-intensity X-ray phenotyping used in Experiment 1: (A) schematic of the system, (B) example of a single image and composite, and (C) illustration of count traits by depth and width of the root system.

A custom X-ray system developed by the PSC was used to image roots growing in polystyrene containers (Fig. 2). The expanded polystyrene containers are nearly transparent in the images at the X-ray energy used (25 kV, 800 µA). Once placed in the X-ray chamber, a computer-controlled positioner moved the plant vertically and horizontally in pre-determined steps to capture eighty 5 cm×5 cm high-resolution X-ray images covering the entire 1 m deep root system. An X-ray imaging system is conceptually similar to a pin-hole camera-based system. The X-ray beam began as a point source and spread out as a cone beam. The exposure time of each X-ray image was ~400 ms. The optical resolution of the system was ~58 µms. The resulting images were gray-scale images, with denser and thicker root tissue being a dark gray to black and very fine diameter root tissue being a light gray.

Each root system was imaged twice—once at the V6 developmental stage and once at the V8 stage. Images were analyzed using RhizoTraits, version 1, a custom software developed by the PSC to extract root traits from X-ray images. RhizoTraits is built off ImageJ (Schneider *et al.*, 2012). Eighty high-resolution X-ray images were combined to create a composite image for analysis of the whole root system (7526×18 194 pixels, 137 MP). A PSC proprietary stochastic-based segmentation algorithm was used to identify root tissue within the images. Quantitative root traits are extracted from the images and for this experiment included (i) total root length and (ii) root system width, at each of 40 transects separated by 25 mm (Table 2; Fig. 2). Analyses were conducted for five root diameter size classes (SCs; Table 2; Fig. 2).

**Table 2.** Plant and root traits measured in controlled environments

Measured plant height (mm)	Height from the top of container to the leaf collar line of the last fully expanded leaf at the vegetative stage of measurement
Size class (SC)	SC1 2900–9860 µm
	SC2 1450–4930 µm
	SC3 725–2465 µm
	SC4 362–1232 µm
	SC5 181–616 µm
TRL (m)	Total root length in meters of all root segments within the defined size class
WidthAtDepth (mm)	Width of the root system at the defined transect depth for roots in the defined size class
CountDensity (*n* mm^–2^)	Number of roots crossing the plane of the defined transect. The area of the plane is given by the cross-sectional area of the container used and the measured width of the root system at the defined depth

Root traits are derived from features extracted from images.

### Soil water uptake contrasting hybrid and plant populations

Previous experiments to evaluate effects of breeding on drought tolerance involved measuring soil moisture content in current plant populations, which may have induced plant to plant variation, and thus overestimated the role of population uniformity in water uptake (Reyes *et al.*, 2015). A field experiment was conducted in Viluco, Chile using the sequence of ERA maize hybrids (Duvick, 2005; Cooper *et al.*, 2020; Table 1) to test the effects of breeding era, plant population, and water stress on soil water uptake (Experiment 2). The experiment included four hybrids from the SX and DX hybrid breeding eras (Table 1), which were replicated eight times in a split-split-plot design, with density as the main plot treatment, irrigation as the subplot treatment, and hybrid as the sub-sub-plot treatment. Two irrigation levels were imposed: low (high) WD treatments received 408 (621) mm of water for both high (10 plants m^–2^) and low (3 plants m^–2^) planting density treatment levels using drip tapes installed 20 cm belowground. The experiment was planted in four-row plots on 7 November 2014 and it was harvested on 1 April 2015. Rows were 4.2 m long and spaced by 0.76 cm. For prior research (Reyes *et al.*, 2015), trenches were excavated to verify the adequacy of four-row plots for studies on water extraction in the Viluco environment. Roots from plants growing in adjacent plots were not observed at the center of the four-row plots where water extraction measurements were made. Soil moisture content was monitored using Time Domain Reflectometry technology (TRIME-PICO IPH/T3, IMKO Micromodultechnik GmbH, Germany). Access tubes were installed in the center of the plot to 1 m where a rock riverbed was reached. In addition to soil moisture measurements, kernel number, ear length, and kernel area per ear were measured using photometric imaging (Cooper *et al.*, 2014b); plant height, leaf number, and size of the ear leaf were measured for two plants per plot. Time to flowering was measured for 10 individual consecutive plants, based on daily observations. For analyses, plants that did not flower after 86 d were assigned a value of 87 d. Leaf area was estimated by length, width, and a 0.79 multiplier. The ratio between water use and leaf area per plant was used to calculate root system efficiency (van Oosterom *et al.*, 2016). The total number of leaves and leaf area of the largest leaf were measured as estimators of plant leaf area (Soufizadeh *et al.*, 2018). Flowering notes and the proportion of barren plants were recorded, based on observations for 10 plants per plot. Yield was measured using imaging methods calibrated for the location (Cooper *et al.*, 2014b).

### Statistical analyses

Total root length and plant height from Experiment 1 were modeled within a linear mixed-effects model framework with the objective to test cross type contrasts between DX and SX hybrids, with named hybrids (*h*) considered as samples taken from a broader population of the hybrid class (*c*),


$$\begin{array}{l} L_{ijkl}=\mu +c_{i}+g_{j}+s_{k}+{\left(cg\right)}_{ij}+{\left(cs\right)}_{ik}+{\left(gs\right)}_{jk}+{\left(cgs\right)}_{ijk}+\delta_{h\left(i\right)}+\epsilon_{ijkl} \end{array}$$ (1)

where *L*_*ijkl*_ is total root length for SC *k* of plant *l* of cross type *i*, and hybrid *h*(*i*), at growth stage *j*. In this model, cross type (DX or SX, *c*_*i*_), growth stage (*g*_*j*_), SC (*s*_*k*_), and all two-way and three-way interactions were considered as fixed effects; hybrid [δ _*h*(*i*)_] served as random effect. The residual term is ∈_*ijkl*_ ~*N*(0, *σ*^2^_*ilk*_), which means that for each level of cross type, growth stage, and size class, a unique variance component *σ*^2^_*ilk*_ is fitted in the mixed model. For the plant height variable, a linear mixed-effects model was fitted,


$$\begin{array}{l} T_{ijl}=\mu +\;c_{i}+g_{j}+{\left(cg\right)}_{ij}+\delta_{h\left(i\right)}+\epsilon_{ijl} \end{array}$$ (2)

where *T*_*ijl*_ represents plant height for plant *l* of cross type *i* at growth stage *j*. Other notations are the same as described for total root length. Cross type, growth stage, and their interaction were considered fixed effects; genotype served as random effect. The residual term of this model is ∈_*ijl*_ ~*N*(0, *σ*^2^_*il*_), where *σ*^2^_*il*_ represents the residual variance term among plants for cross type *i* at growth stage *j*, allowing each level of cross type and growth stage to have a unique residual variance component.

To test for heterogeneity of plant to plant variation between SX and DX hybrids under different growth stages or root SCs, a likelihood ratio method was applied using two nested mixed-effects models. For the plant height trait, a cross type fixed effect and a hybrid (nested in cross type) random effect are included in both models.


$$\begin{array}{l} y_{il}=\mu +\;c_{i}+\delta_{h\left(i\right)}+\epsilon_{il} \end{array}$$ (3)

For the full model (M1), the residual variance parameter depends on the level of cross type, ∈_*il*_ ~*N*(0, *σ*^2^_*i*_). For the reduced model (M2), a single residual variance parameter is used for all observations, ∈_*il*_ ~*N*(0, *σ*^2^). The *P*-value of the likelihood ratio test was calculated as


$$\begin{array}{l} P\{X_{\;\Delta \;df}^{2}>2\times \left[loglik\left(M1\right)-loglik\left(M2\right)\right]\} \end{array}$$ (4)

where ∆d*f*=d*f*(M1)–d*f*(M2). A similar approach was implemented to test plant to plant variation among cross types for root-level traits. For a given growth stage, the full model (M1),


$$\begin{array}{l} y_{ikl}=\mu +\;c_{i}+s_{k}+{\left(cs\right)}_{ik}+\delta_{h\left(i\right)}+\epsilon_{ikl} \end{array}$$ (5)

where, cross type, SC, and its interaction were considered fixed effects. Hybrid was considered as random effect. For root-level trait models, the residual term contributes to plant to plant variation. In the full model (M1), the residual variance parameter depends on the specific level of cross type and root SC, ∈_*ikl*_ ~*N*[0, *σ*^2^_*ik*_(∈)]. In contrast, the reduced model (M2) considering the residual parameter depended only on the root class level, ∈_*ikl*_ ~*N*[0, *σ*^2^_*k*_(∈)].

The models to test plant to plant variation for traits that vary with depth (d) conditional on growth stage, M1 and M2, were extended to include the variable depth and interaction with cross type and SC as fixed effects:


$$\begin{array}{l} y_{ikml}=\mu +\;c_{i}+s_{k}+d_{m}+{\left(cs\right)}_{ik}+{\left(cd\right)}_{im}+{\left(sd\right)}_{km}+{\left(csd\right)}_{ikm}+\delta_{h\left(i\right)}+\epsilon_{ikml} \end{array}$$ (6)

where hybrid was considered as a random effect. Just like the models of root-level traits, M1 has a residual variance parameter depending on cross type and root size ∈∈_*ikml*_ ~*N*[0, *σ*^2^_*ik*_(∈)]. For the reduced model, the residual variance parameter does not vary between cross types, ∈_*ikml*_ ~*N*[0, *σ*^2^(∈)]. To test the heterogeneity of plant to plant variation between SX and DX hybrids for two growth stages for a certain root SC, a similar method was applied with the underlying factor as root SC. In this case, SC was replaced with growth stage in the previous models.

The width of the root system was modeled as a function of depth using a non-linear mixed-effects model, with the underlying non-linear function as a gamma-Ricker function:


$$\begin{array}{l} w=a_{1}\times d^{\gamma }\times e^{-a_{2}\times d} \end{array}$$ (7)

where width (*w*) is set as the response variable and depth (*d*) as the explanatory variable. For all three parameters *a*_1_, *a*_2_, and γ, growth stage (*j*), cross type (DX or SX, *i*), and their interaction were considered fixed effects, and hybrid and plant (*l*) as random effects. Taking γ as an example, the mixed effect model is


$$\begin{array}{l} \gamma_{ijl}=\mu +c_{i}+g_{j}+{\left(cg\right)}_{ij}+\delta_{h\left(i\right)}+p_{l(i)\;\;\;} \end{array}$$ (8)

where the hybrid random effect δ _*h*(*i*)_ ~*N*[0, *σ*^2^_*i*_(δ)] and the plant random effect *p*_*l*(*i*)_ ~*N*[0, *σ*^2^_*i*_(δ)] . Parameters and fitted curves were estimated for each root SC (SC3, SC4, and SC5).

Reproductive and vegetative traits from Experiment 2 were analyzed within a generalized linear mixed-effects model framework to test for differences between cross type DX and SX. For the traits with continuous numeric values, a Gaussian model with identity link was used. For the traits with count or fraction values (e.g. proportion of barren plants), a binomial model with logistic link was used. In the generalized linear mixed-effects model, cross type, plant population, location, and their interactions were considered fixed effects, while field spatial factor defined as row and columns, and named hybrid nested in cross type (Table 1) were considered random effects. The proportion of barren plants was modeled as


$$\begin{array}{l} y_{h\left(i\right),\;jkxy}∼Binomial[\left(N,\;p_{h\left(i\right),jkxy}\;\right)\; \end{array}$$ (9)

$$\begin{array}{ll} log\left(\frac{p_{h\left(i\right),jkxy}}{1-p_{h\left(i\right),jkxy}}\right)\;=\mu +c_{i}+b_{j}+l_{k}+{\left(cb\right)}_{ij}\; & \\ +{\left(cl\right)}_{ik}+{\left(bl\right)}_{jk}+{\left(cbl\right)}_{ijk}+\alpha_{x,l}+\beta_{y,l}+\delta_{h\left(i\right)} \end{array}$$ (10)

where *c*_*i*_ is cross type (DX or SX) effect, *b*_*j*_ is plant population effect, *l*_*k*_ is irrigation treatment effect, (*cb*)_*ij*_, (*cl*)_*ik*_, (*bl*)_*jk*_, and (*cbl*)_*ijk*_ are the two factor and three factor interaction effects between cross type, population, and irrigation, α _*x*,*l*_ and β _*y*,*l*_ are row and column effects at each irrigation treatment with α _*x*,*l*_ ~*N*[0, *σ*^2^_*l*_(α)] and β _*y*,*l*_ ~*N*[0, *σ*^2^_*l*_(β)], δ _*h*(*i*)_ is the named hybrid random effect with δ _*h*(*i*)_ ~*N*[0, *σ*^2^_*i*_(δ)], and *σ*^2^_*l*_(α), *σ*^2^_*l*_(β), and *σ*^2^_*i*_(δ) are variance parameters for the three random effects in the model.

A generalized additive model with integrated smoothness was applied to analyze the effect of cross type, plant population, and total depth (800 mm or 1000 mm) on the temporal dynamics of soil water content for Experiment 2. The dependent variable *y* was total available soil water (mm) and the independent variable was days after planting (*x*),


$$\begin{array}{l} y_{ijk}\left(x\right)\;∼\;\mu +c_{i}+p_{j}+d_{k}+{\left(cp\right)}_{ij}+{\left(cd\right)}_{ik}+{\left(\;pd\right)}_{jk}\;\\ +{\left(cpd\right)}_{ijk}+f_{1}\left(x;c_{i}\right)+f_{2}\left(x;p_{j}\right)+f_{3}(x;d_{k}) \end{array}$$ (11)

where hybrid cross (DX or SX) (*c*_*i*_), plant population (*p*_*j*_), total depth (*d*_*k*_), and their interactions served as the parametric terms. The functions *f*_1_(*x*;*c*_*i*_), *f*_2_(*x*;*p*_*j*_), and *f*_3_(*x*;*d*_*k*_), are the smoothing terms by cross type, plant population, and total depth, respectively. Cubic regression spline bases with dimensions of 20 were used to fit the smoothing function *f*_1_, *f*_2_, and *f*_3_. All the non-parametric smoothing terms estimated here were centered at 0. In this model, each parametric term represents the overall magnitude of a certain fixed effect, while each smoothing term represents the pattern of the curve under each specific level of the corresponding factor.

All the linear mixed models and generalized linear mixed models were estimated using Asreml version 3 (Gilmour *et al.*, 2009). The non-linear mixed-effect models were fitted using R package ‘nlme’ version 3.1-144 (Pinheiro *et al.*, 2020). The generalized additive models were fitted using the R package ‘mgcv’ (Wood, 2011).

## Results and discussion

### Root system architecture changed with long-term selection for yield in maize

**Fig. 3.**
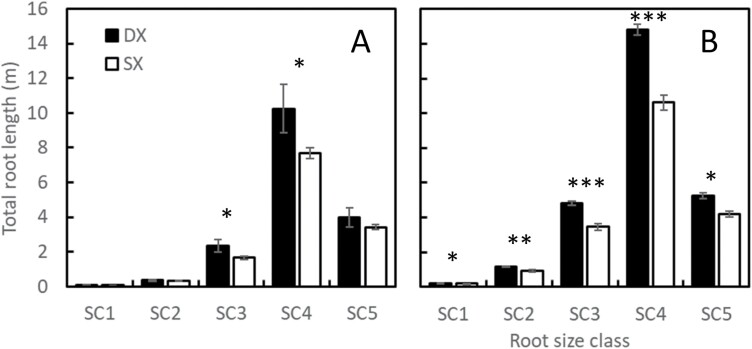
Best linear unbiased estimators for total root length (m) between DX and SX hybrids by root size class (SC; Table 2) when six (A) and eight (B) leaves were fully expanded from Experiment 1. *P<0.1, **P<0.05, ***P<0.01.

**Fig. 4.**
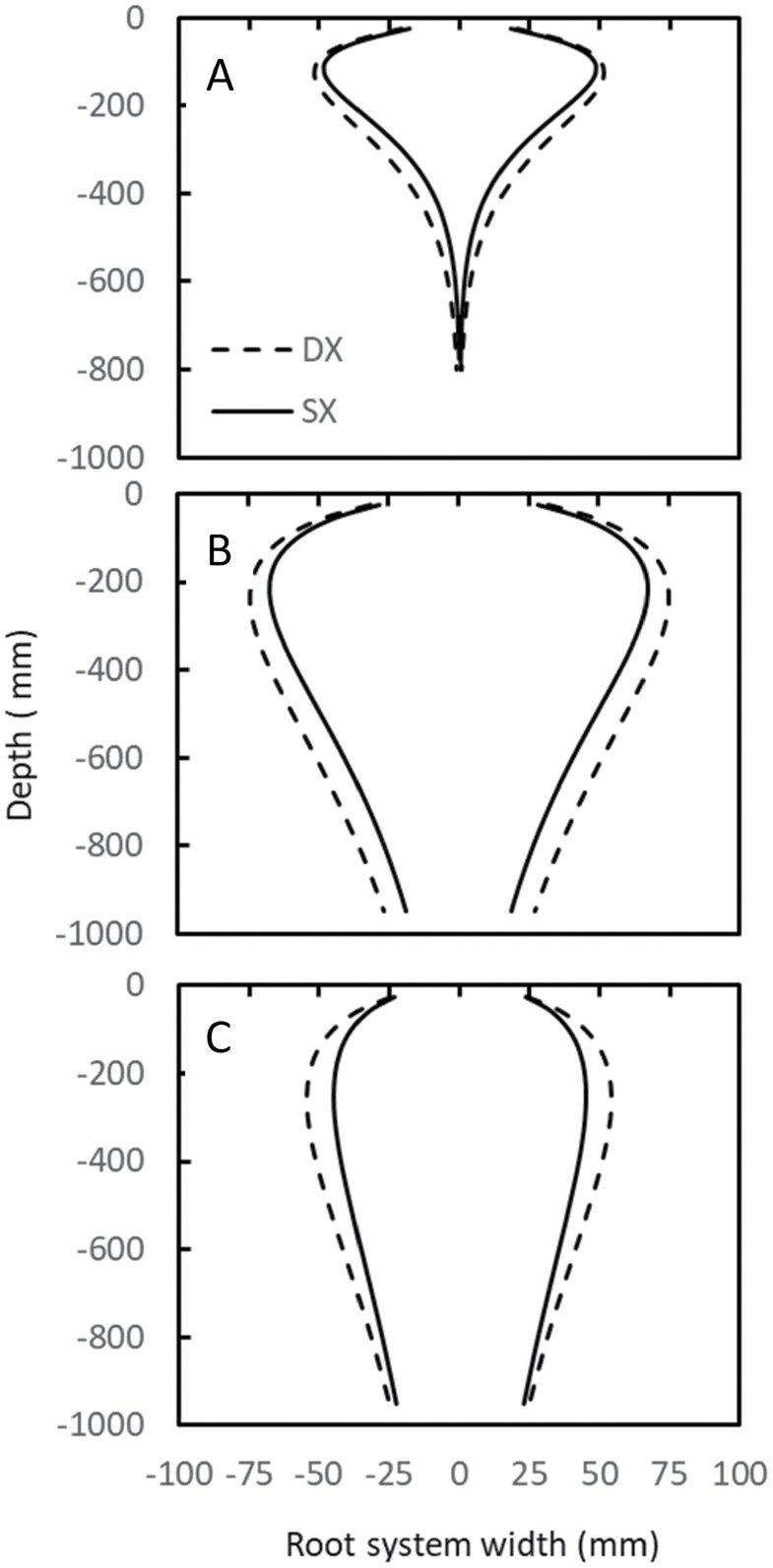
Best linear unbiased estimators for root systems width measured using X-ray PSC technology for root SC3 (A, 725 –2465 µm), SC4 (B, 362–1232 µm), and SC5 (C, 181– 616 µm) at stage of development V8 from Experiment 1. Predictions for root system width (W) by each depth (d) are centered. Г functions are: W_SX,SC=3_=(**0.57**±0.19)×d^**1.37**±0.085^×e^(**0.0119**±0.0007×d)^, W_DX,SC=3_=(**1.31**±0.33)×d^**1.143**±0.06^×e^(**0.0092**±0.0005×d)^, W_SX,SC=4_=(6.77±0.95)× d^0.68±0.03^×e^(0.0031±0.0002×d)^, W_DX,SC=4_=(8.80±1.08)×d^0.63±0.03^×e^(0.0027±0.0002×d)^, W_SX,SC=5_=(**10.59**±2.42)×d^0.47±0.05^×e^(0.0019±0.00024×d)^, W_DX,SC=5_=(**8.36**±1.69)×d^0.56±0.04^×e^(0.0022±0.0002×d)^. Significant differences (*P*<0.05) between DX and SX hybrids in function parameters are indicated in bold.

A maize hybrid set spanning a century of breeding (ERA hybrids; Table 1) was used as a case study to test the hypothesis that water capture underpins crop improvement in maize. This set comprises hybrids commercialized since 1920 that were widely adopted by farmers of the time. The sequence starts with the open-pollinated Reid Yellow Dent and ends with AQUAmax® drought-tolerant germplasm (Duvick 2005; Cooper *et al.*, 2020). Maize SX and DX hybrids were exposed to contrasting water treatments and plant population densities to determine genetic gain in water uptake and yield. Root architecture was measured using X-ray technology (Fig. 2). Consistent with theoretical predictions (Hammer *et al.*, 2009; Fig. 1A, B), we show that older DX hybrids had significantly greater root length than modern SX hybrids (Fig. 3B). The largest effect of long-term selection manifested on the upper soil layers (Fig. 4). Modeling RSA using a gamma-Ricker function, we were able to establish that root systems of DX hybrids were generally wider than those of SX hybrids, and the difference was significant (*P*<0.05) for roots of diameters between 725–2465 µm and 181–616 µm (Fig. 4A, C).

Following the principles of Darwinian agriculture (Denison *et al.*, 2003), the observed differences between DX and SX hybrids in total root length (Fig. 3) and width (Fig. 4) could have been caused by plant to plant variation in root size due to genetic segregation (Duvick, 2001) and intraspecific competition (Daynard and Muldoon, 1983). No significant differences were detected for plant height. In contrast, significant plant to plant variation was detected for root traits, which were measurable in total root length for SC2, SC3, and SC4, root system width for all SCs except SC2, and density for all SCs (Tables 2, 3). However, root system width is an indicator of the outer bound of root occupancy of a given volume of soil but not how effectively this volume is explored by the root system. The root length to width ratio (LWR) provides a metric to assess plausible changes from DX to SX hybrids in their capacity to explore occupied volumes. The root LWR calculated from total root length and width, and for SC3, SC4, and SC5 (Table 2) at V8, were 0.17, 0.08, and 0.04 cm cm^–2^ for DX hybrids, and 0.17, 0.08, and 0.03 cm cm^–2^ for SX hybrids. We show that neither the allometry between root SCs nor the efficiency by which roots explore an occupied volume have changed between the DX and SX breeding eras.

**Table 3.** Plant to plant variation characterized by SEs of the trait measured using X-ray phenotyping at development stage V8

Size class	Cross type	TRL (m)		Width (mm)		Density (*n* mm^–2^)	
		SE	*P*-value	s.e.	*P*-value	SE	*P*-value
SC1	DX	0.11		19.5		0.04	
	SX	0.11	0.5	17.33	<0.01	0.05	<0.01
SC2	DX	0.54		37.47		0.05	
	SX	0.58	<0.05	35.24	0.16	0.04	<0.01
SC3	DX	2.11		52.64		0.16	
	SX	1.57	<0.01	48.20	<0.01	0.11	<0.01
SC4	DX	4.56		45.89		0.10	
	SX	3.44	<0.01	51.78	<0.01	0.14	<0.01
SC5	DX	1.39		55.74		0.20	
	SX	1.47	0.5	57.98	<0.10	0.30	<0.01

### Water uptake remained unchanged over eras of maize breeding

**Fig. 5.**
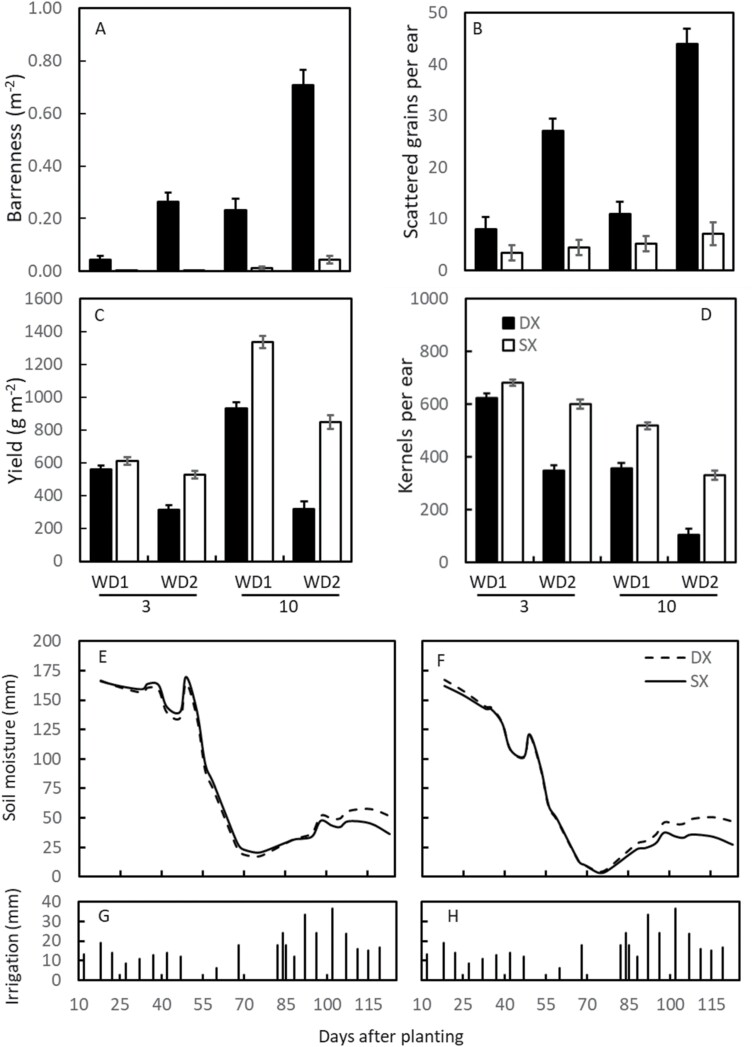
Best linear unbiased estimators for contrasts between double cross (DX) and single cross (SX) maize hybrids grown in Chile (Experiment 2) for barren plants (A), grains scattered in the cob (B), yield (C), kernels per ear (D), grown under 3 plants m^–2^ and 10 plants m^–2^, and two irrigation regimes (WD1=621 mm and WD2=408 mm), and temporal dynamics of plant-available soil water (mm) measured in WD2 in a 1 m soil column and at 3 plants m^–2^ (E) and 10 plants m^–2^ (F). Irrigation amounts are displayed in (G) and (H).

Results from the root morphology and water uptake experiments indicate that selection improved root system efficiency but not total soil water capture. SX hybrids had a smaller RSA per plant when measured in growth chambers (Fig. 4), but they captured the same volume of water as the DX hybrids (Fig. 5) despite the similar leaf area (Table 4). While patterns of water use differed between hybrid types (*P*<0.05; Fig. 5E, F), the capacity to capture water from the soil, estimated by the change in water content between 18 d and 74 d after planting, was similar for SX and DX hybrids and plant populations (Fig. 5E, F). SX and DX hybrids used water at rates of 2.7 mm d^–1^ and 2.6 mm d^–1^, and of 2.8 mm d^–1^ and 2.9 mm d^–1^ when grown under low and high plant population density, respectively (Fig. 5E, F). Differences between hybrid types occurred during the late grain-filling period possibly due to the capacity of modern hybrids to maintain their leaf area under stress (Duvick, 2001, 2005). Dividing the rate of water use by the average size of the ear leaf (Table 2), an estimator of plant canopy size (Soufizadeh *et al.*, 2018), and by total root length, an estimator of root system size (van Oosterom *et al.*, 2016), here we show that DX hybrids have lower root system efficiency than SX hybrids (0.00012 d m^–1^ versus 0.00016 d m^–1^).

**Table 4.** Best linear unbiased estimators and SEs for main effect of crop and plant traits measured in Chile (Experiment 2) under two plant population densities (3 and 10 plants m^–2^) and irrigation treatments (WD1=621 mm and WD2=408 mm) for double (DX) and single (SX) hybrids

Population	Irrigation	Hybrid group	TLN	Size of ear leaf	Time to anthesis	ASI
(plants m^-2^)			(count)	(cm^2^)	(d)	(d)
3	WD1	DX	20.0±0.6	974±26	73.89±0.86	0.37±0.78
		SX	19.4±0.5	1062±27	70.92±1.17	–1.25±0.94
	WD2	DX	20.2±0.5	924±25	72.38±0.54	4.82±1.04
		SX	19.4±0.5	1023±26	71.44±0.96	–0.38±1.17
10	WD1	DX	19.6±0.5	964±25	72.00±0.60	1.87±0.78
		SX	19.3±0.5	1023±26	69.75±1.00	–0.81±0.94
	WD2	DX	20.0±0.5	888±25	72.44±0.62	4.69±1.67
		SX	19.5±0.5	957±26	72.06±1.01	0.56±1.75

Size of ear leaf and TLN are the size of the largest leaf and the total number of leaves on the main stem, ASI is anthesis–silking interval.

The difference in soil moisture between low and high density plant populations was 17±2.8 for DX hybrids and 13±2.8 mm for SX hybrids when the soil moisture reached a minimum value. This result suggests that plant population is the main controlling factor of root occupancy and water capture, and that there was enough water in the soil column to quantify differences in soil water capture due to variation in RSA if differences were present (Fig. 5E, F). At low plant population density, between 17 mm and 20 mm of water was measurable in the soil. This water could have been utilized by the hybrid group with larger root systems or canopies. However, soil water content was not significantly different between the DX and SX groups when the soil moisture was at the minimum under high density (–0.8±3.8 mm; Fig. 5E, F). No differences were observed despite DX hybrids presumably having larger root systems based on the X-ray study (Figs 3, 4).

### Yield improvement in maize was driven by enhanced reproductive resilience

In contrast to results shown for water capture, yield and yield components were significantly higher for SX than DX hybrids across treatments (*P*<0.001). Yield of DX hybrids decreased with increasing barrenness, and barrenness increased with increasing density and WD (Table 4; Fig. 5A, B). Yield of both DX and SX hybrids decreased with decreasing kernels per ear (Fig. 5C, D). Kernels per ear decreased with increasing anthesis–silking interval (ASI) [*y=*528(±48)–67.26(±19.03)×*x*, *r*=0.81]. The lower absolute correlation between kernels per ear and ASI relative to yield and ASI (|*r*|=0.81 versus |*r*|=0.58) is associated with compensation between kernel weight and number within fertile ears (Borrás *et al.*, 2004; Messina *et al.*, 2019). The high association between barrenness and ASI for DX hybrids, and the observation of scatter grains (Fig. 5B) indicates that protandry induced by WD (Hall *et al.*, 1982; Fuad Hassan *et al.*, 2008) was a major driver underpinning a reduction in kernels per ear. Because both SX and DX groups reached anthesis at the same time for an irrigation and planting density treatment (Table 4), and with the same soil water content (Fig. 5E, F), we can rule out that differences in stress were due to timing of reproductive stages and soil water content. The higher ASI and barrenness observed for DX than for SX hybrids indicates that protandry for DX hybrids was long enough to miss at least part of the pollination window (Messina *et al.*, 2019). In addition, WD caused significant reductions in kernels per ear, which were larger in DX than in SX hybrids (Fig. 5D), which implies differences between hybrid types in tolerance to water deficit beyond those explained by protandry alone (Fig. 5C, D; Table 4). Significant differences between plant populations and hybrid types in yield and yield components indicate variation in stress tolerance unrelated to water capture (Table 4; Fig. 5C, D), such as sequential floret development (Oury *et al.*, 2016), reduced sensitivity of silk elongation to drought (Fuad Hassan *et al.*, 2008; Messina *et al.*, 2019), maintenance of carbon metabolism (Zinselmeier *et al.*, 1999; McLaughlin and Boyer, 2004), and resource allocation to the ear (Edmeades *et al.*, 1993).

### Yield gain from iterative genetic and agronomic optimization

We conclude that selection did not operate to increase water capture per plant and that the higher reproductive resilience in SX hybrids is not a consequence of improved water capture as postulated (Hammer *et al.*, 2009; Messina *et al.*, 2011) and reported before for a cohort of SX hybrids (Reyes *et al.*, 2015). Instead, yield improvement since the commercialization of DX hybrids in maize is related to improved water capture due to higher planting rates that translate into higher aerial mass and yield. The differential response measured as ASI, kernels per ear, and yield between SX and DX hybrids when exposed to the same level of WD (Fig. 5E, F) provides unequivocal evidence that genetic improvement of yield precedes changes in RSA. We propose a non-dichotomous view, whereby selection for yield led to improvements in reproductive resilience, which in turn enabled changes in the structure of the plant community including plant density. Changes in agronomic practices such as plant population density could have led to changes in optimal root architecture, which further contributed to exposing genetic variation for RSA traits and genetic gain for yield. RSA adapted to increasingly crowded stands by decreasing the root system angle, increasing the efficiency of water uptake, and increasing reproductive resilience through shifts in carbon allocation. Reduced metabolic costs associated with smaller root systems, such as the phenotypes with reduced branching (Lynch *et al.*, 2014; Zhan *et al.*, 2015), could contribute to carbon reallocation. The lower total root length, higher occupancy of small roots, equal or higher total plant leaf area, and constant water uptake suggest that SX hybrids have higher root system efficiencies measured on a per root length basis than DX hybrids. The observation that both SX and DX hybrids capture the same amount of soil water at low plant population density suggests that genetic improvement operated towards optimizing RSA for improved efficiency of water capture. While the underpinning for the improved RSA efficiency is unknown, adaptive root growth response to water availability via ARF7, or ethylene-mediated response to soil compaction, are hypotheses worth testing (Orosa-Puente *et al.*, 2018; Pandey *et al.*, 2021).

The reduction in RSA width, rather than being a cause of improved water capture as proposed (Hammer *et al.*, 2009; Messina *et al.*, 2011), is a contributor to improved root system efficiency and stress tolerance through shifts in carbon allocation to the ear and increased water capture through increased plant population. By decreasing reproductive failure under stress conditions, enhanced reproductive resilience increases yield stability (Messina *et al.*, 2018) and can explain observations that selection operated to reduce genotype×environment interactions for yield in temperate maize (Gage *et al.*, 2017). Results conform to regional analyses indicating yield improvement across levels of WD (Lobell *et al.*, 2014). Drought is an important component of the target population of environments where yield is >2000 g m^–2^, which is the yield level estimated by Cooper *et al.* (2020) at which water becomes non-limiting to yield. Hence, we propose that improved reproductive resilience, as shown here, underpins the reported yield gains in the vast majority of the US corn belt (Lobell *et al.*, 2014). Because water capture has not changed between SX and DX hybrids, the results are also consistent with the reported changes in relative sensitivities to WD, and the observation that maize yield association with soil plant-extractable water is more marked in the drier regions of the US corn belt (Lobell *et al.*, 2020). The relative importance of soil water capture over reproductive resilience increases with increasing WD.

The feedback between genetics and agronomy, and the evidence that the impact of root phenomics and selection on yield within breeding programs have been slow (Tracy *et al.*, 2020), brings into question the feasibility of ideotype breeding in maize for root systems as proposed before (Meister *et al.*, 2014). However, improved phenotyping capabilities as shown here and elsewhere (Zhu *et al.*, 2011; Kuijken *et al.*, 2015; Atkinson *et al.*, 2019), and recent advances in understanding root elongation, efficiency, and responsiveness to water content, soil compaction, and plant density (Lynch, 2013; Lynch *et al.*, 2014; York *et al.*, 2015; Zhan *et al.*, 2015; Orosa-Puente *et al.*, 2018; Pandey *et al.*, 2021; Schneider *et al.*, 2021) can help accelerate the impact of root biology on yield improvement. In contrast to previous cycles of selection, a germplasm improved for reproductive resilience will express changes in RSA as improvements in yield under WD. However, there are limitations on the speed at which one can integrate root phenotypes within breeding programs due to the sequential and iterative nature of co-selection and adaptation (Cooper *et al*., 2014 ). Exploring adjacent spaces in the adaptation landscape (Messina *et al.*, 2011), whereby shifts in traits are tested as hypotheses within the genotype×management systems, can be a more productive approach to accelerate yield improvement. Using crop modeling and genomic prediction, it is possible to identify maize crosses that vary in tolerance to drought and plausible root elongation rate. This hypothesis could be tested within a breeding program using advanced root phenomics. With a clear definition of breeding objectives and precision phenomics, prediction methodologies that integrate quantitative genetics and agronomy models (Messina *et al.*, 2018, 2020; Cooper *et al.*, 2020; Peng *et al.*, 2020) offer a path to accelerate genetic gain for multiple outcomes. It may be possible to simultaneously improve yield, carbon sequestration in the soil (Kell *et al.*, 2012), and yield stability under drought by rebalancing growth relationships between roots, shoots, and reproductive structures.

## Data Availability

The data and scripts used for analyses can be made available through https://openinnovation.corteva.com/ upon reasonable request for public research purposes and project evaluation.

## Abbreviations

DX: double cross
LWR: root length to width ratio
PSC: Phenotype Screening Corporation
RSA: root systems architecture
SC: size class for roots
SX: single cross
WD: water deficit
